# Metallicity in a Holstein-Hubbard Chain at Half Filling with Gaussian Anharmonicity

**DOI:** 10.1038/s41598-017-03985-2

**Published:** 2017-06-19

**Authors:** Ch. Uma Lavanya, I. V. Sankar, Ashok Chatterjee

**Affiliations:** School of Physics, University of Hyderabad, Central University P. O., Hyderabad, 500046 Telangana India

## Abstract

The Holstein-Hubbard model with Gaussian phonon anharmonicity is studied in one-dimension at half filling using a variational method based on a series of canonical transformations. A fairly accurate phonon state is chosen to average the transformed Holstein-Hubbard Hamiltonian to obtain an effective Hubbard model which is then solved using the exact Bethe - ansatz following Lieb and Wu to obtain the ground state energy, the average lattice displacement and the renormalized parameters. The Mott-Hubbard criterion, local spin moment and the von Neumann entropy (which is a measure of quantum entanglement) are calculated to determine the ground state phase diagram which shows that the width of the metallic phase flanked by the SDW and CDW phases increases with increasing anharmonicity at low and moderate values of anharmonicity but eventually saturates when the anharmonicity becomes substantially large.

## Introduction

There have been several studies on the cuprate superconductors in which the origin of high temperature superconductivity has been attributed to the electron-phonon (*e-p*) interaction^1–12^. Plakida^5^ has argued that a strong coupling between electrons and phonons and hence the high value of *T*_*c*_ can be obtained due to instability of the ion-lattice at a moderate *e-p* coupling. Alexandrov^6^ has shown that the ordinary *e-p* interaction narrows the polaron band resulting in the high temperature superconducting behavior. In the context of *e-p* interaction in these materials, the Holstein-Hubbard (HH) model seems to be the most suitable model^8, 9, 12^. The ground state (GS) properties and the phase diagram of the extended Holstein-Hubbard model have been studied, among others, by Sil *et al*.^9^ and by Sankar and Chatterjee^10^. The main bottle-neck in the polaronic mechanism of high *T*_*c*_ superconductivity is that this mechanism requires for high - *T*_*c*_ a large *e-p* interaction, which however is expected to drive the system to the insulating charge density wave (CDW) state. On the other hand, at small *e-p* interaction, one expects the on-site electron-electron (*e-e*) Coulomb correlation to dominate in these systems leading to an insulating spin density wave (SDW) state. Thus according to the conventional wisdom, one would expect only a direct insulator-insulator transition in these materials as the *e-p* interaction is increased. Recently, however, Takada and Chatterjee (TC)^13^ have shown within the framework of the one-dimensional HH model at half filling that it is possible to have an intervening metallic phase at the cross-over region of the SDW-CDW transition. A further widening of the intermediate metallic phase has been shown by Krishna and Chatterjee (KC)^14^ by using a better variational calculation^15^. Subsequently, several other studies have also shown the evidence of this metallic phase^16–27^. This problem has recently been studied by Sankar and Chatterjee^28^ by calculating the von Neuman entropy which gives a measure of the Quantum Entanglement (QE) and hence the metallicity and the quantum nature of the phase transition associated with the SDW-CDW transition. The existence of the reasonable evidence of the intermediate metallic phase at the SDW-CDW cross-over region notwithstanding, this issue is not conclusively settled because the calculation of TC^13^ has been restricted to a very simple phonon state and the calculation of KC^14^ is only marginally better.

In the above investigations, phonons have been considered to be harmonic while in reality the ion-ion potential in a solid should also contain anharmonic terms giving rise to phonon-phonon interactions and hence a finite life-time for phonons. The effects of anharmonic phonons on electronic properties are normally very small and therefore they are generally neglected. In recent years, however, several investigations have shown that phonon anharmonicity can produce some profound effects on the electronic properties as well, particularly in the high-*T*_*c*_ superconductors^29–33^. In this context, anharmonic vibration of the apex oxygen atoms in cuprates has attracted particular attention and its effects have been studied in terms of various models for the anharmonic phonons. Also, the competition between superconductivity and CDW has been studied in the HH model with quartic anharmonic contribution to the phonon potential energy^34–36^. Chatterjee and Takada (CT)^37^ have shown using the HH model that the phonon anharmonicity can make the polarons even more mobile and broadens the metallic phase. In this investigation, however, the phonon anharmonicity has been considered only up to the quartic power. Konior^8^ has considered a Gaussian anharmonic polaronic model and showed that the band narrowing factor is less rapidly decaying function of the *e-p* interaction strength. The advantage with the Gaussian anharmonicity model is that it contains phonon anharmonicity up to infinite orders and is expected to give convergent results under all conditions, a property that is lacking in cubic and quartic anharmonicities^37^. In the present work, our aim is to study the effect of the Gaussian phonon anharmonicity on the metallic phase at the CDW-SDW crossover region in a HH model using the Mott criterion, the local moment value and the quantum entanglement with an accurate phonon state.

## The Model

The Holstein-Hubbard model is described by the Hamiltonian

1 $$H={H}_{el}+{H}_{ph}+{H}_{el-ph},$$

with

1a $${H}_{el}=-t\sum _{\langle ij\rangle \sigma }{c}_{i\sigma }^{\dagger }{c}_{j\sigma }+U\sum _{i}{n}_{i\uparrow }{n}_{i\downarrow },$$

1b $${H}_{ph}=\hslash {\omega }_{0}\sum _{i}{b}_{i}^{\dagger }{b}_{i}+{\lambda }_{ap}\sum _{i}{e}^{-\gamma {({b}_{i}^{\dagger }+{b}_{i})}^{2}},$$

1c $${H}_{el-ph}=g\sum _{i\sigma }{n}_{i\sigma }({b}_{i}^{\dagger }+{b}_{i}),$$

where ${c}_{i\sigma }^{\dagger }({c}_{j\sigma })$ is the creation (annihilation) operator for an electron with spin *σ* at site *i*, *t* refers to the nearest-neighbor hopping integral, ${n}_{i\sigma }(={c}_{i\sigma }^{\dagger }{c}_{i\sigma })$ is the number operator for an electron of spin *σ* at site *i*, *U* represents the onsite *e-e* Coulomb interaction energy, ${b}_{i}^{\dagger }({b}_{i})$ is the creation (annihilation) operator for a phonon of dispersionless frequency *ω*_0_ at site *i*, *λ*_*ap*_ and *γ* denote respectively the strength and range of the lattice potential and *g* gives the onsite *e-p* interaction strength.

## Formulation

### GS energy and average lattice displacement

We first perform the variable-displacement Lang-Firsov (VDLF) transformation^38–41^ with the generator: ${R}_{1}=\frac{{g}^{{\rm{^{\prime} }}}}{\hslash {\omega }_{0}}\sum _{i,\sigma }{n}_{i\sigma }({b}_{i}^{\dagger }\,-\,{b}_{i})$ where *g*′ is a variational parameter. We assume that *g*′ is of the form: $g^{\prime} =g\,\eta =\sqrt{\alpha }\,\eta$, where *α* is the dimensionless *e-p* coupling constant and *η* essentially gives a measure of the lattice deformation. In the convensional Lang-Firsov (LF) approach^38^, one chooses *η* = 1 so that *g*′ = *g* and obtains the GS energy by averaging the transformed Hamiltonian with respect to the zero-phonon state which is a good enough approximation for strong *α* in the anti-adiabatic limit. For the entire coupling range, however, a lower GS energy can be obtained by optimizing *η*. Furthermore, one assumes within the framework of VDLF approach that the phonon coherence coefficient depends linearly on *n*_*iσ*_. However, it is possible to have an *n*_*iσ*_-independent phonon coherence that may lower the energy and that can be achieved by the Takada-Chatterjee transformation^13^ with the generator ${R}_{2}=\sum _{i}{h}_{i}({b}_{i}^{\dagger }-{b}_{i})$, where *h*_*i*_ is another variational parameter. The above two transformations can be accomplished by a single transformation with a generator, ${R}_{12}=\sum _{i}[{h}_{i}+\sqrt{\alpha }\eta (\frac{1}{\hslash {\omega }_{0}}-\frac{{h}_{i}}{\sqrt{\alpha }})]\,({b}_{i}^{\dagger }\,-\,{b}_{i})$. When *η* = 1, one has the usual LF transformation which is valid in the anti-adiabatic approximation and *η* = 0 gives the *n*_*iσ*_-independent coherent state transformation. Thus the two-transformations together with a variable *η* (0 < *η* *<* 1) encompass the entire parameter space of *t* and *ω*_*0*_, from the anti-adiabatic limit (*η* = 1) to the adiabatic limit (*η* = 0). We shall assume that *h*_*i*_ = *h* for all *i* which is a reasonable approximation since all sites can be considered identical. This is also consistent with the site-independent choice for *g*′. So far we have assumed the phonon sub-system to be coherent and completely neglected the phonon-correlation effect which may play an important role as is well known from polaron physics. In the language of field theory, an electron is the source of phonons and when an electron emits a phonon, it recoils back due to the finite phonon momentum, and while recoiling the electron can emit another phonon, particularly in the case of reasonable electron-phonon interaction, and in that case those two successively emitted virtual phonons will be correlated. This correlation leads to the squeezing of the coherent phonon state and it has been shown^42^ that it also reduces the Holstein reduction factor considerably and consequently makes the polaron bandwidth larger leading to a higher mobility of the polarons which is more favourable for a metallic state. The presence of phonon anharmonicity in the Hamiltonian also introduces a finite life - time for phonons and this finite life - time effect can be included in the phonon dynamics by squeezing the phonon state. The squeezing of the phonon sub-system can be achieved by performing a unitary transformation with a generator ${R}_{3}=\alpha ^{\prime} \sum _{i}(\,{b}_{i}{b}_{i}-{b}_{i}^{\dagger }{b}_{i}^{\dagger })$, where *α*′ is the squeezing parameter to be obtained variationally. It may also be pointed out that this transformation also takes into account some effects of the phonon anharmonicity and therefore incorporates the dynamics of the anharmonic phonons i. e., the finite life - time effects. To obtain the effective electronic Hamiltonian we average the fully transformed Hamiltonian with respect to the phonon state^8^: $|{\varphi }_{ph}\rangle ={\sum }_{n=0}^{M}{c}_{n}{\phi }_{n}(x)$, where *φ*_*n*_(*x*) is the eigenfunction of the *n*-th excited state of a simple harmonic oscillator of frequency *ω*_0_ and the expansion coefficients *c*_*n*_’s are obtained variationally. We start with *M* = 0 and keep increasing the value of *M* till we get a convergent result. The effective electronic Hamiltonian is finally obtained as

2 $$\begin{array}{rcl}{H}_{eff} & = & {\varepsilon }_{eff}\sum _{i\sigma }{n}_{i\sigma }-{t}_{eff}\sum _{\langle ij\rangle \sigma }{c}_{i\sigma }^{\dagger }{c}_{j\sigma }+{U}_{eff}\sum _{i}{n}_{i\uparrow }{n}_{i\downarrow }\\  &  & +N\hslash {\omega }_{0}[\frac{{e}^{4\alpha ^{\prime} }}{4}{S}_{2}-\frac{{e}^{-4\alpha ^{\prime} }}{4}{S}_{3}-\frac{1}{2}-h{e}^{2\alpha ^{\prime} }{S}_{1}+{h}^{2}]+N{\lambda }_{ap}{E}_{1},\end{array}$$

with

2a $$\begin{array}{ccc}{\varepsilon }_{eff} & = & -\frac{(2g-{g}^{^{\prime} }){g}^{^{\prime} }}{\hslash \,{\omega }_{0}}+(g-{g}^{^{\prime} })[{e}^{2{\alpha }^{^{\prime} }}{S}_{1}-2h]+{\lambda }_{ap}({E}_{2}-{E}_{1}),\\ {t}_{eff} & = & t{F}^{2},\\ {U}_{eff} & = & U-\frac{2{g}^{^{\prime} }}{\hslash \,{\omega }_{0}}(2g-{g}^{^{\prime} })+{\lambda }_{ap}({E}_{3}-\,2{E}_{2}+{E}_{1}),\\ {S}_{i} & = & \sum _{k,l\,=\,0}^{M}{c}_{kl}{\int }_{-{\rm{\infty }}}^{{\rm{\infty }}}{e}^{-{y}^{2}}{\xi }_{i}(y){H}_{k}(y){H}_{l}(y)dy,\,\\ F & = & \,\,\sum _{k,l\,=\,0}^{M}{c}_{kl}{e}^{-\frac{{a}^{2}}{4}}{\int }_{-{\rm{\infty }}}^{{\rm{\infty }}}{e}^{-{y}^{2}}{H}_{k}(y+\frac{a}{2}){H}_{l}(y-\frac{a}{2})dy,\\ {E}_{i} & = & \sum _{k,l=0}^{M}{c}_{kl}{\int }_{-{\rm{\infty }}}^{{\rm{\infty }}}{e}^{-{y}^{2}-\gamma {(\sqrt{2}y{e}^{2{\alpha }^{^{\prime} }}-2h-{\zeta }_{i})}^{2}}{H}_{k}(y){H}_{l}(y)dy\end{array}$$

where ${c}_{kl}={c}_{k}{c}_{l}\sqrt{1/{2}^{k+l}k!l!\pi \,},\,{\xi }_{1}=\sqrt{2}y,\,{\xi }_{2}\,=2{y}^{2},\,{\xi }_{3}=2({y}^{2}-2l-1),y=\sqrt{\frac{{\omega }_{0}}{\hslash }}x,a=\frac{1}{\hslash {\omega }_{0}}\sqrt{2}{g}^{^{\prime} }{e}^{-2{\alpha }^{^{\prime} }}$, *ζ*_1_ = 0,$\,{\zeta }_{2}=2g^{\prime} /\hslash {\omega }_{0}$ and *ζ*_3_ = 4*g*′/*ħω*_0_ for *i* = 1, 2 and 3. The on-site Coulomb interaction and the hopping integral are renormalized as *U*_*eff*_ and *t*_*eff*_. Following Lieb and Wu^43^, we solve *H*_*eff*_ exactly by the Bethe - ansatz technique at half filling to obtain the GS energy per electron (*ε*) as:

3 $$\begin{array}{ccc}\varepsilon  & = & \frac{{e}^{4{\alpha }^{{\rm{^{\prime} }}}}}{4}{S}_{2}-\frac{{e}^{-4{\alpha }^{{\rm{^{\prime} }}}}}{4}{S}_{3}-\frac{1}{2}-h{e}^{2{\alpha }^{{\rm{^{\prime} }}}}{S}_{1}+{h}^{2}+{\lambda }_{ap}{E}_{1}-J\\  &  & +\frac{{U}_{eff}-|{U}_{eff}|}{4}-{\int }_{0}^{{\rm{\infty }}}\frac{4{t}_{eff}{J}_{0}(\xi ){J}_{1}(\xi )d\xi }{\xi [1+exp(\xi \frac{|{U}_{eff}|}{2\,{t}_{eff}}\,)]},\end{array}$$

where

3a $$J=(2g-g^{\prime} )g^{\prime} -(g-g^{\prime} )[{e}^{2\alpha ^{\prime} }{S}_{1}-2h]+{\lambda }_{ap}{E}_{1}-{\lambda }_{ap}{E}_{2}.$$

To obtain the GS energy we numerically minimize Eq. (3) with respect to the parameters *η*, *h*, *α*′ and *c*_*n*_’s. The average lattice displacement is given by

4 $$\langle {x}_{i}\rangle ={e}^{2\alpha ^{\prime} }({S}_{1}/\sqrt{2}-\sqrt{2}g^{\prime} -\sqrt{2}h).$$

### Local spin moment

The average electron spin moment per site can be written as

5 $${L}_{0}=\frac{1}{N}\sum _{i}\langle {S}_{i}^{2}\rangle =\frac{3}{4}-\frac{3}{2N}\sum _{i}\langle {n}_{i\uparrow }{n}_{i\downarrow }\rangle $$

which on using (3) yields

5a $${L}_{0}=\frac{3}{4}-\frac{3}{2}\frac{d\varepsilon }{dU}.$$

A non-zero value of *L*_*0*_ at the SDW-CDW cross-over region would confirm the existence of an intervening metallic phase in that region. It has been suggested^13–15^ that for uncorrelated electrons the value of *L*_*0*_ is equal to 3/8 (=0.375). It has been furthermore argued^16^ that the value of *L*_*0*_ varies between 3/8 (=0.375) (band limit) and 3/4 (=0.75) (atomic limit) for a pure Hubbard model.

### Entanglement Entropy

Since the entanglement entropy is useful to analyze the properties of correlated quantum phase transitions^44–52^, it would be interesting to study it for HHM. Consider the four possible states |0〉, |↑〉, |↓〉 *and* |↑↓〉. The entanglement entropy can be measured by calculating the von Newmann entropy: *E*_*ϑ*_ = −*Tr*(*ρ*_*r*_ *log*_2_ *ρ*_*r*_), where *ρ*_*r*_ is the reduced density matrix which can be written as

6 $${\rho }_{r}={\omega }_{e}|0\rangle \langle 0|+{\omega }_{\uparrow }|\uparrow \rangle \langle \downarrow |+{\omega }_{\downarrow }|\downarrow \rangle \langle \uparrow |+{\omega }_{\uparrow \downarrow }\,|\uparrow \downarrow \rangle \langle \uparrow \downarrow |,$$

where *ω*_↑↓_ = 〈*n*_*i*↑_ *n*_*i*↓_〉 ≡ *ω* gives the double occupancy, *ω*_↑_ = *ω*_↓_ = (*n*/2) − *ω*_↑↓_ and *ω*_*e*_ = 1 − *ω*_↑_ − *ω*_↓_ − *ω*_↑↓_. The entanglement entropy *E*_*ϑ*_ can be determined using the Hellmann-Feynman theorem: (∂*ε*/∂*U*) = 〈*n*_*i*↑_ *n*_*i*↓_〉.

## Results and Discussion

**Figure 1:**
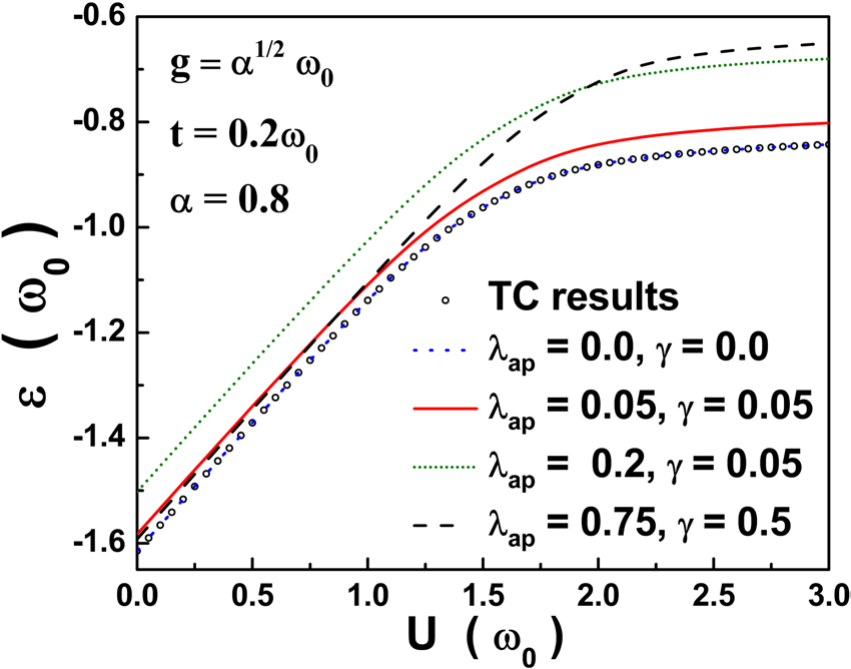
GS energy (*ε*) vs. on-site *e-e* correlation strength *U*.

For numerical calculation, we consider three anharmonic cases (i) *λ*_*ap*_ = 0.05, *γ* = 0.05 (ii) *λ*_*ap*_ = 0.2, *γ* = 0.05 and (iii) *λ*_*ap*_ = 0.75, *γ* = 0.5 and set *ћω*_*0*_ = 1. The case (i) corresponds to low anharmonicity, case (ii) represents the moderate level anharmonicity and the case (iii) is for large anharmonicity. We consider the anti-adiabatic regime and set *t* = 0.2 *ω*_0_ throughout the present work. Figure 1 shows the variation of the GS energy per site of the system as a function of on-site coulomb interaction strength (*U*) for both harmonic and anharmonic cases. For *U* ≤ 1, anharmonicity does not seem to have much effect, but at large *U*, anharmonicity does enhance the energy. For the harmonic case (*λ*_*ap*_ = *γ* = 0) we obtain exactly the TC results^13^.

**Figure 2:**
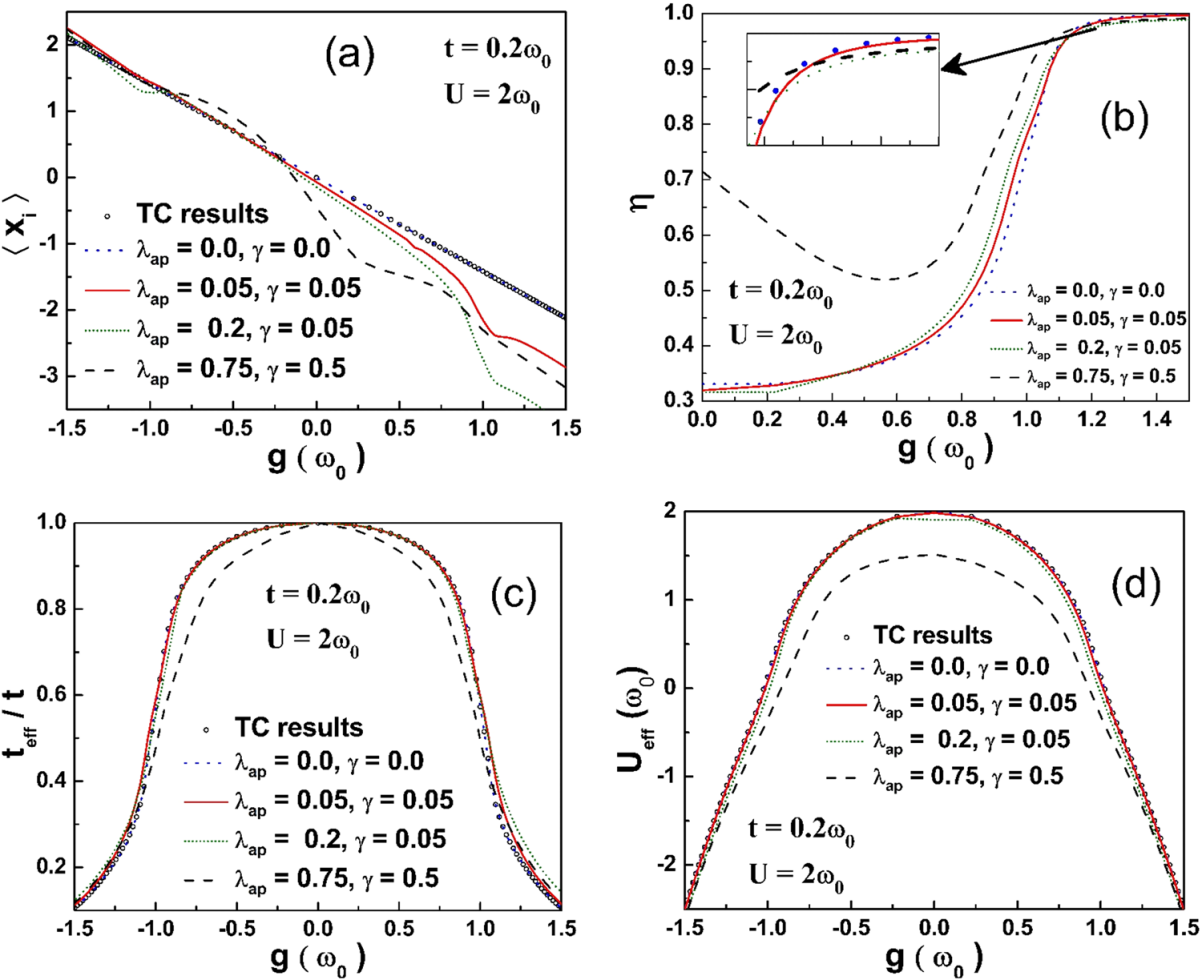
(**a**) 〈*x*_*i*_〉 as a function of *g* for different values of *λ*_*ap*_ and *γ*. (**b**) *η* vs. *g*. (**c**) *t*_*eff*_/*t* as a function of *g*. (**d**) *U*_*eff*_ as a function of *g*.

Figure 2(a) illustrates the behavior of the average lattice displacement 〈*x*_*i*_〉 as a function of the *e-p* interaction strength *g*. 〈*x*_*i*_〉 decreases with increasing *g*. For the harmonic case *λ*_*ap*_ = 0 = *γ*, the present results agree well with the TC results^13^ imparting a fair amount of confidence in the TC results. For a large value of anharmonicity, 〈*x*_*i*_〉 behaves in a complicated way and looks asymmetric in *g*. Figure 2(b) shows the plot of optimized *η* vs. *g*. It is interesting to note that there is a qualitative difference in the behavior of *η* for a sufficiently large anharmonicity, namely, *η* goes through a minimum before saturating to the strong-coupling value. Figure 2(c) shows that the band narrowing factor decreases rapidly with increasing *g*. In the case of large anharmonicity, band-narrowing factor diminishes even at a faster rate. Figure 2(d) shows that as *g* increases, the effective on-site *e-e* interaction (*U*_*eff*_) decreases. In the presence of anharmonicity, *U*_*eff*_ is reduced even further. For the harmonic case, $\langle {x}_{i}\rangle =-\sqrt{2}g$, where $g=\surd \alpha$, *α* being the dimensionless *e-p* coupling. As the anharmonicity is increased, the value 〈*x*_*i*_〉 deviates from the harmonic case to a higher magnitude for positive *g*. This change is associated with the decrease in *U*_*eff*_, *t*_*eff*_/*t* and *L*_0_ which in turn is due to the enhancement in the optimized value of *η* from its corresponding harmonic value. After *g* ≈ 1.1, anharmonicity reduces *η* which in turn enhances *t*_*eff*_/*t* leading to the formation of mobile polarons. This reduction also depends on the competition between the *e-e* and *e-p* interaction strengths. If the anharmonicity is substantially large, it can make the *e-p* interaction strong enough to overcome the *e-e* Coulomb repulsion.

**Figure 3:**
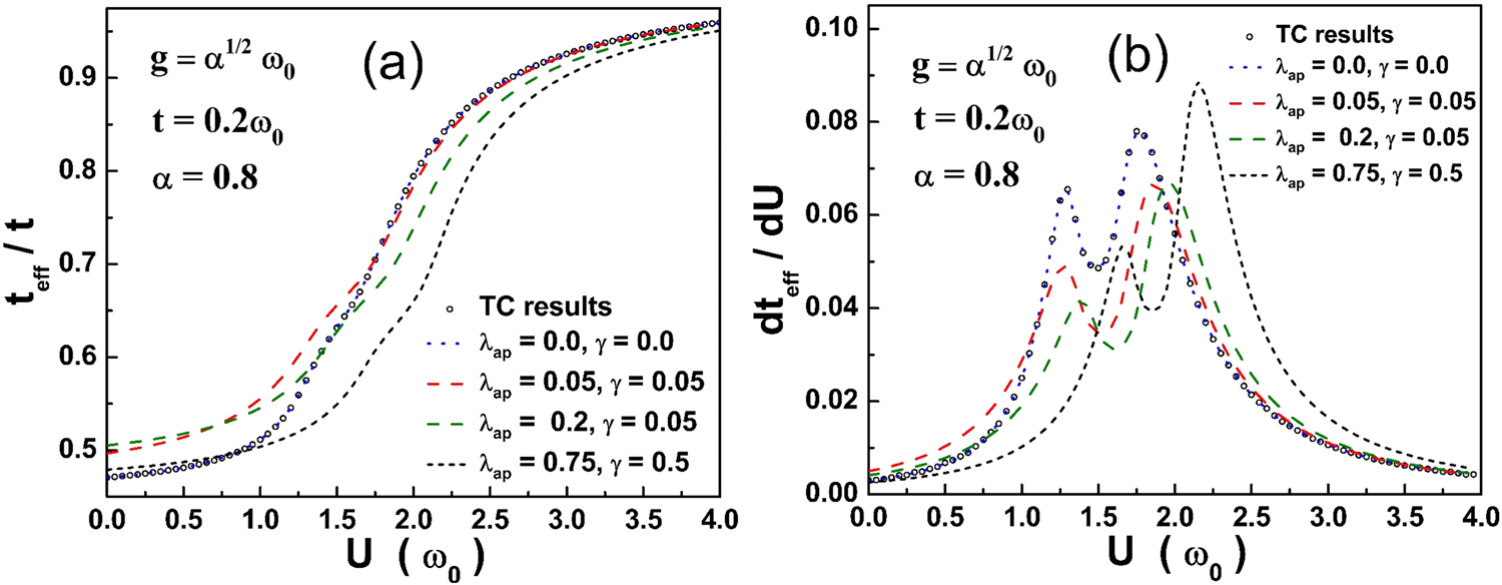
(**a**) *t*_*eff*_/*t* vs. *U*; (**b**) *dt*_*eff*_/*dU* vs. *U*.

In Fig. 3(a). *t*_*eff*_/*t* is plotted against *U* for three anharmonic cases. The harmonic case is compared with TC results and it agrees well. The value of *t*_*eff*_/*t* reaches 1 as *U* becomes strong. The plot of *dt*_*eff*_/*dU* vs. *U* in Fig. 3(b) shows a double-peak structure as in the harmonic case but the peak heights are higher for stronger anharmonicity. Also the peaks shift to the right with increasing anharmonicity because the phonon anharmonicity strengthens the *e-p* interaction and therefore a larger *U* is required to cause the transition displayed by the peak structure.

**Figure 4:**
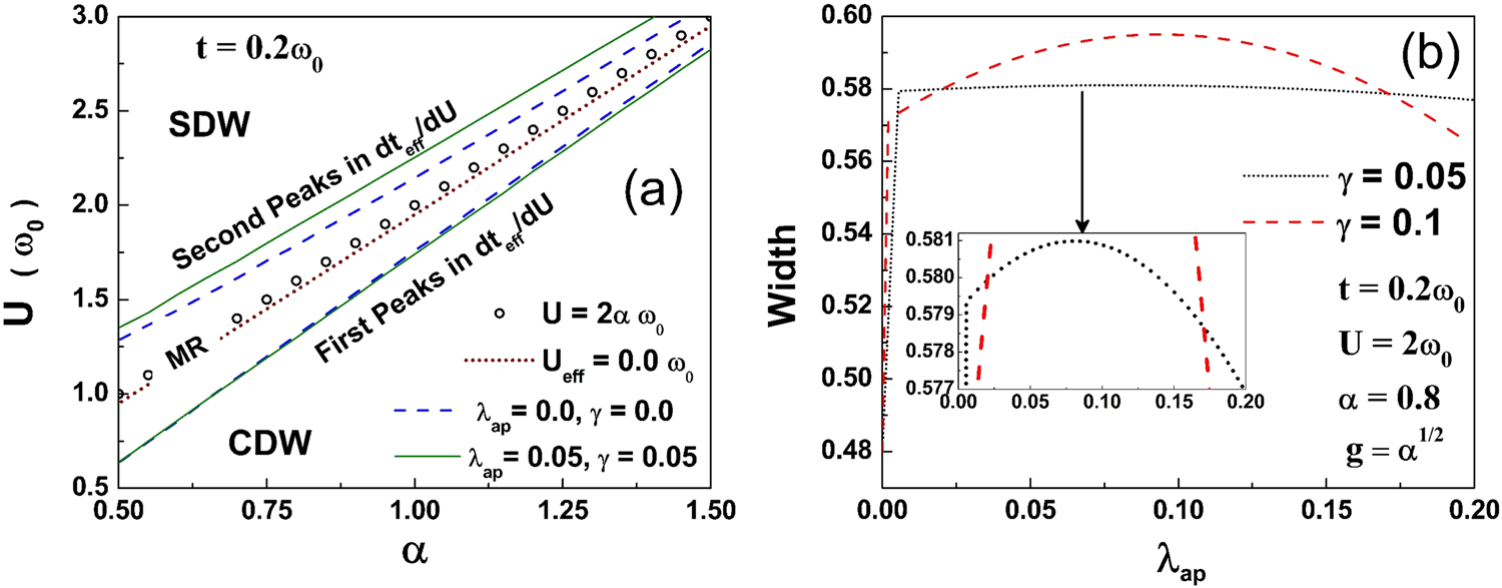
(**a**) Phase diagram in *α*−*U* plane determined from the peaks in *dt*_*eff*_/*dU*. MR represents metallic region (**b**) Peak-to-peak width from *dt*_*eff*_/*dU*-plot as a function of *λ*_*ap*_ for two values of *γ*.

The phase diagram in the *α*−*U* plane as determined by the peaks in dt_*eff*_/dU is shown in Fig. 4(a). The effect of anharmonicity on the metallic phase is shown in Fig. 4(b) for two cases. The width of the metallic phase for the harmonic system is 0.48 (in units of *ω*_0_). As the Gaussian anharmonicity is introduced, the width of the metallic phase increases sharply to about 0.58 (in units of *ω*_0_). As the anharmonicity is increased further, the width continues to increase, attains a maximum and then decreases with further increase in the anharmonicity.

**Figure 5:**
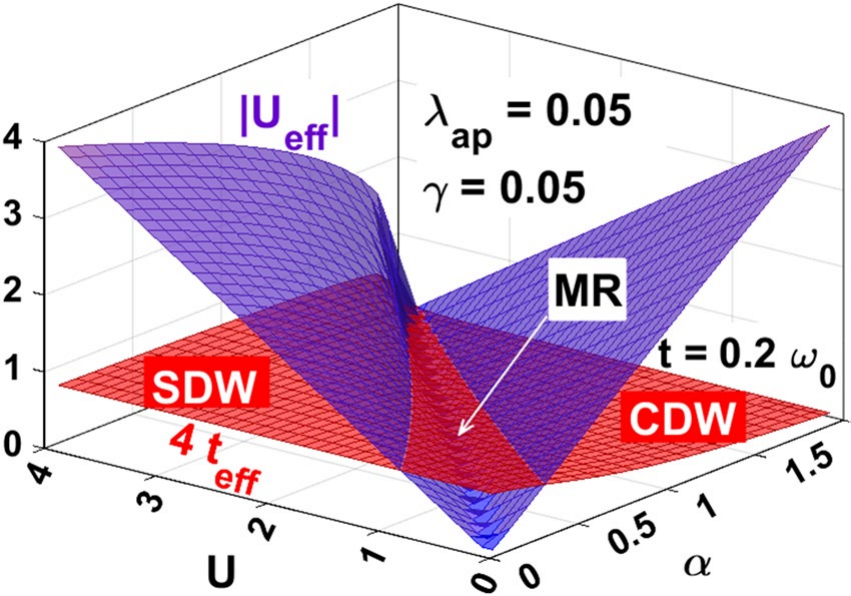
A 3D picture showing the behavior of |*U*_*eff*_| (blue) and 4*t*_*eff*_ (red) with *U* and *α*.

Figure 5 gives a 3-dimensional picture of the metallic phase that exists at the CDW-SDW crossover region for a given *λ*_*ap*_ and *γ*. The red surface in between the two blue surfaces satisfies the condition: $4{t}_{eff}\,\gtrapprox |{U}_{eff}|$ and thus corresponds to a metallic phase. On the left side of the metallic region, *U*_*eff*_ is positive, which corresponds to the SDW GS, while on the right side of the metallic region, *U*_*eff*_ is negative, which corresponds to the CDW GS. Thus we conclude that as *α* is increased, the system makes a transition from the antiferromagnetic SDW GS state to the bipolaronic CDW GS state through a metallic phase. This result is important because it suggests that one can manipulate the material parameters in such a way that even if the *e-p* interaction is large, the system GS can still be in a metallic state and become superconductive at low temperatures. It is clear from Fig. 4(b) that the moderate anharmonicity is most favorable from the point of view of superconductivity.

**Figure 6:**
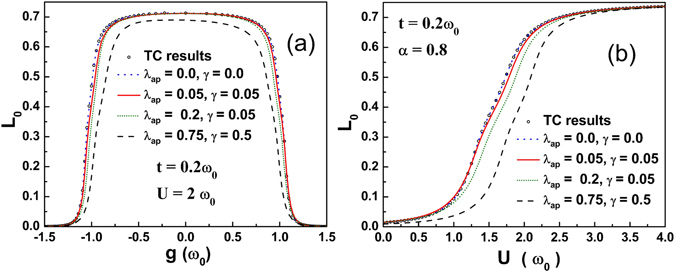
(**a**) Variation of local spin moment *L*_0_ with *g*. (**b**) Variation of *L*_0_ with *U* for different values of *λ*_*ap*_ and *γ*.

Figure 6(a) shows the variation of the local spin moment (*L*_0_) as a function of *g* with different sets of values of *λ*_*ap*_ and *γ*. One can notice that *L*_*0*_ has a very weak dependence on *g* for lower anharmonic systems, beyond which *L*_*0*_ falls off very rapidly to zero. This is because as *g* is increased by a very small amount, the hopping probability of the electron to the neighboring site increases only marginally leading to a very slight reduction in *L*_*0*_. As *g* exceeds a certain critical value, *L*_*0*_ decreases rapidly to zero. The reason is simple. For large g the net *e-e* interaction becomes attractive and as a result two electrons can occupy a particular site making *L*_*0*_ equal to zero. *L*_*0*_ is suppressed significantly for the large anharmonic case while for the lower and moderate anharmonic cases *L*_*0*_ is suppressed slightly. Figure 6(b) shows the variation of *L*_*0*_ as a function of *U*. As *U* increases, electrons repel one another resulting in a higher value of *L*_*0*_ at a particular site. Up to *U* ≈ 1, *L*_*0*_ does not show much discernible change, but for $1\lesssim U\lesssim 2.2$, it undergoes a significant monotonic rise. A further increase in *U* leads *L*_*0*_ to essentially saturate.

**Figure 7:**
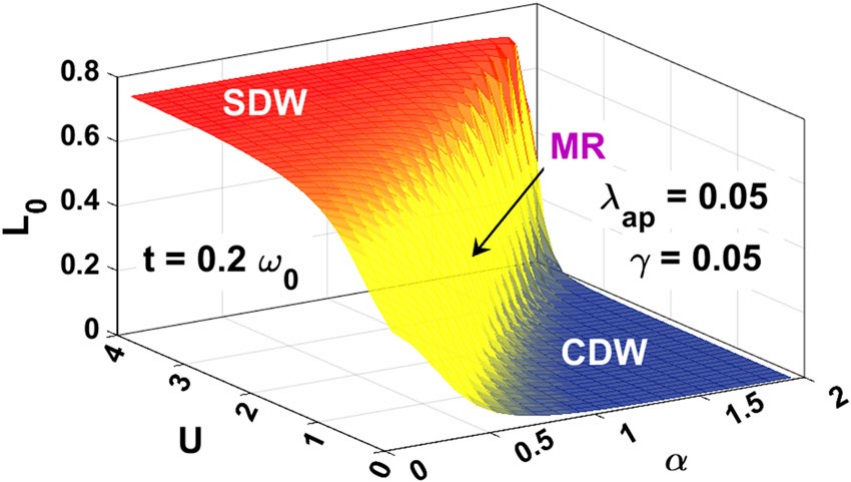
*L*_*0*_ vs. *U* and *α* for *λ*_*ap*_ = 0.05, *γ* = 0.05 and *t* = 0.2 *ω*_*0*_.

**Figure 8:**
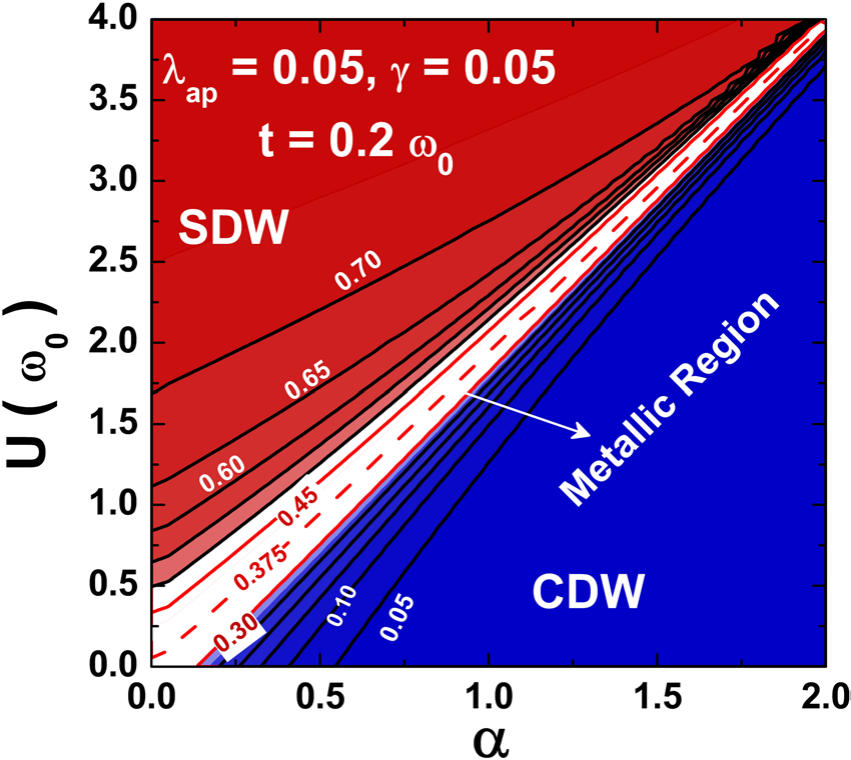
Contour plots of *L*_*0*_ in *α*-*U* plane.

Figure 7 gives the surface plot of *L*_*0*_ as a function of *α* and *U* and Fig. 8 gives the contour plots of *L*_*0*_ in the *α*-*U* plane. For a completely uncorrelated electron gas, *L*_*0*_ = 0.375 which is indeed the value we see in the intermediate phase in Figs 7 and 8. This provides yet another evidence of the existence of an intervening metallic phase at the CDW-SDW crossover region.

**Figure 9:**
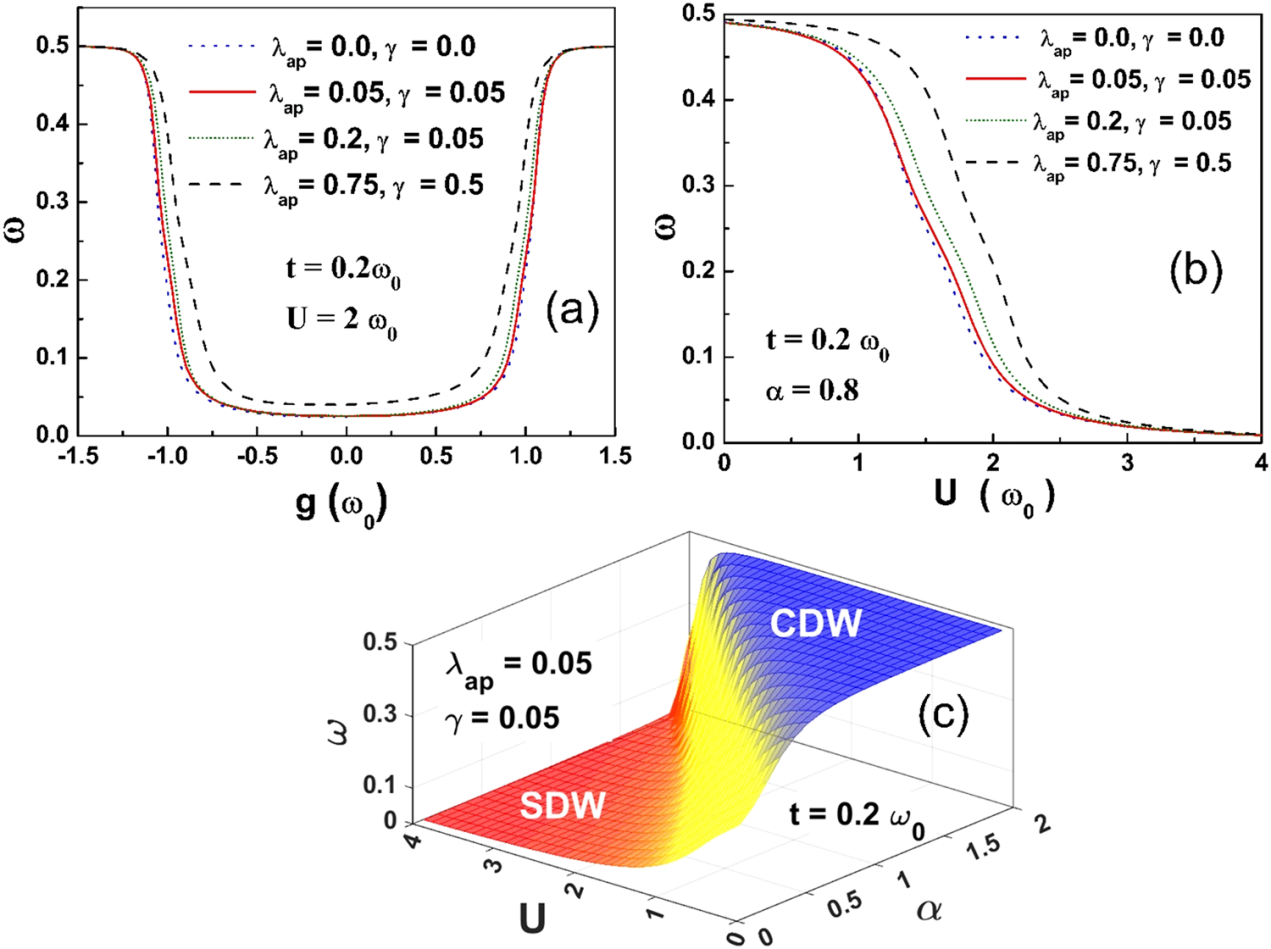
(**a**) Double occupancy *ω* as a function of *g*. (**b**) *ω* as a function of *U*. (**c**) *ω* as a function of *α* and *U*.

Figure 9(a) shows the variation of double occupancy, *ω*, as a function of *g* for several values of *λ*_*ap*_. For small positive values of *g*, *ω* is low which is indicative of an SDW phase. As *g* is increased, *ω* increases rapidly and beyond a critical value of *λ*_*ap*_ it saturates to a constant indicating the formation of bipolarons corresponding to the CDW phase. The double occupancy *ω* also increases, albeit marginally, with increasing anharmonicity, of course, at low *g* values. Figure 9(b) gives the variation of *ω* with respect to *U*. For small values of *U*, the effective on-site *e-e* interaction strength, *U*_*eff*_ becomes negative. This leads to a large value of *ω* implying a bipolaron or a CDW phase. As *U* is increased, *U*_*eff*_ becomes positive leading to a reduction in *ω*. The double occupancy *ω* tends to zero which corresponds to an SDW state above a certain value of *U*. Figure 9(c) shows double occupancy as a function of *α* and *U*.

**Figure 10:**
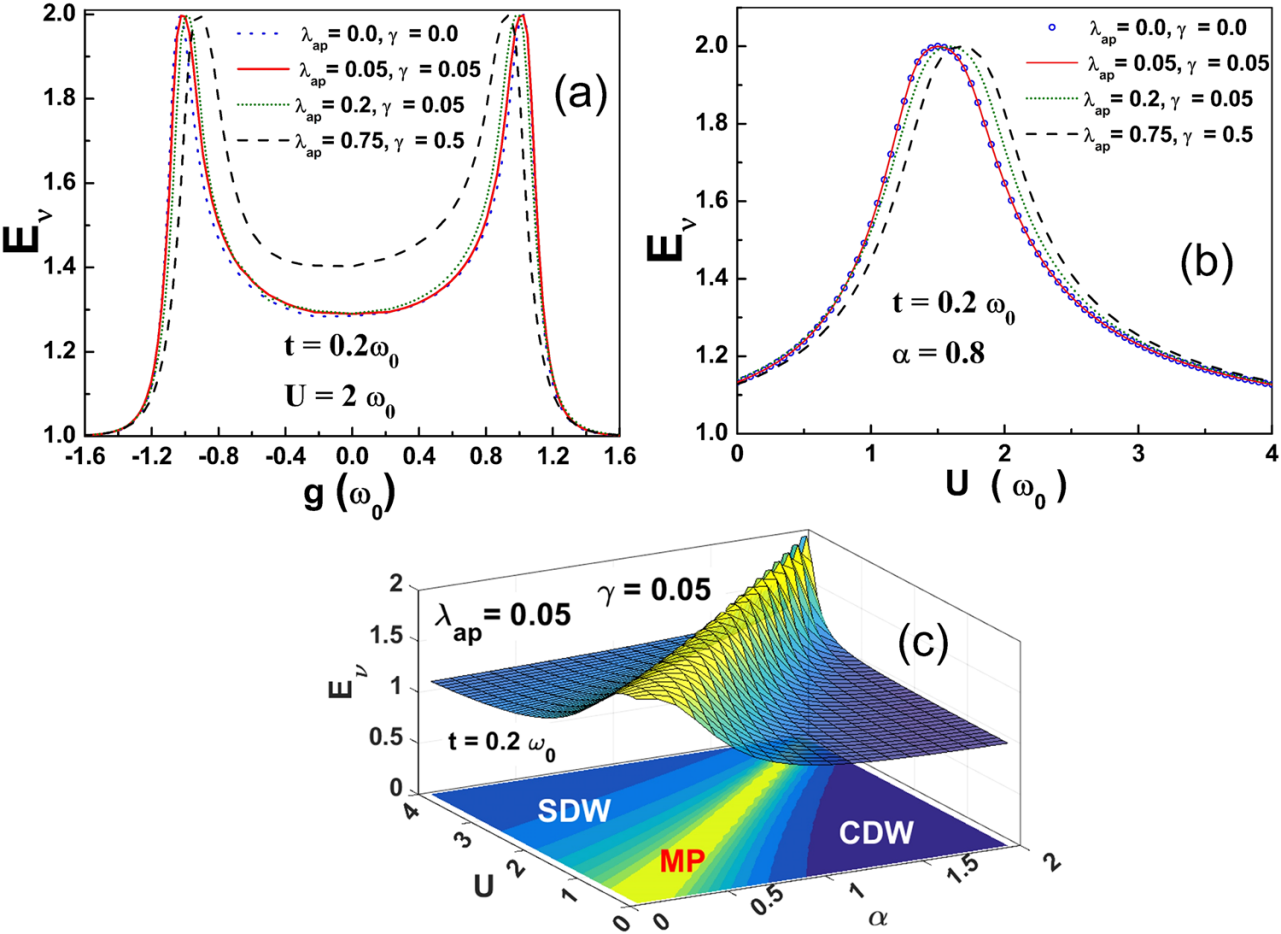
(**a**) Quantum Entanglement (*E*_*ϑ*_) as a function of g. (**b**) *E*_*ϑ*_ as a function of *U*. (**c**) *E*_*ϑ*_ on the *α*-*U* plane with its contour map.

Figure 10(a). describes the variation of von Neumann entropy (*E*_*0*_) (which is a measure of QE) with respect to *g*. One can see that *E*_*0*_ is symmetric for the harmonic case while it is asymmetric for anharmonic cases. As *g* increases from zero, QE increases slowly and develops a peak and then falls rather sharply with a further increase in *g*. As we increase the anharmonicity the peak shifts towards the lower values of *g*. The more is the entanglement, the more the interactions between the electrons. The peak in the entanglement indicates the metallic phase. Figure 10(b) shows that QE has a peak at a certain value of *U* which is a clear indication of the presence of the metallic phase at the SDW-CDW crossover region. One can also observe that the peak shifts towards the higher values of *U* as the anharmonicity increases. Figure 10(c) shows the plot of *E*_*0*_ as a function of *α* and *U*. QE has a unique peak at the region which satisfies the criteria of metallic phase as shown in the Fig. 5.

**Figure 11:**
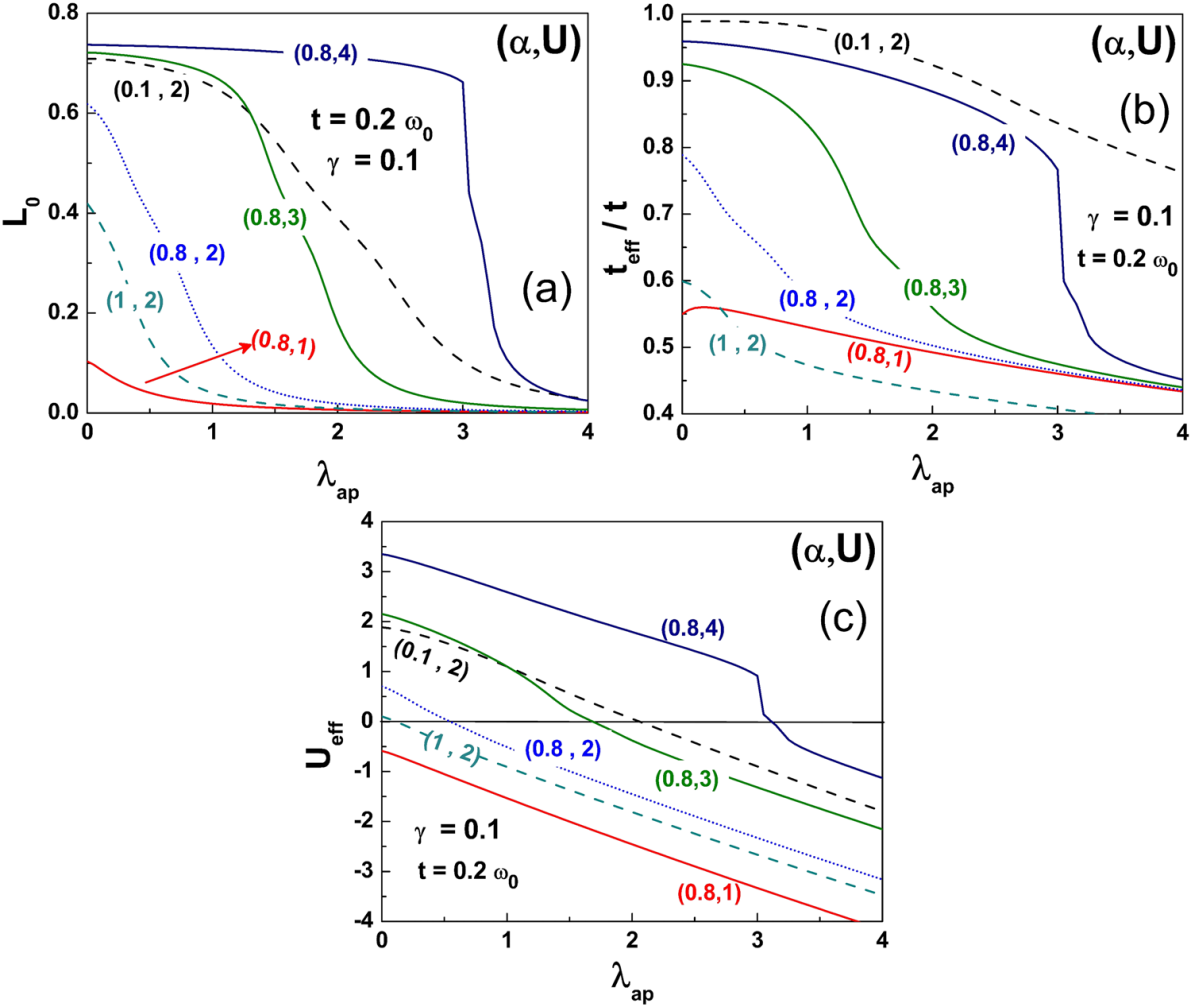
(**a**) Local spin moment *L*_*0*_ as a function of the strength of the Gaussian anharmonicity *λ*_*ap*._ (**b**) Renormalized inter - site electron hopping term t_*eff*_/t as a function of *λ*_*ap*_. (**c**) Renormalized on-site *e-e* interaction strength as a function of *λ*_*ap*_.

In order to study the effect of anharmonicity on the phase transition, we have plotted *L*_*0*_, *t*_*eff*_/*t* and *U*_*eff*_ as a function of *λ*_*ap*_ in Fig. 11(a–c) respectively. A first glance on these figures gives the information that all the mentioned quantities on the ordinate decrease when the anharmonicity is introduced in the system. A close look on these plots suggests the following points. (i) In Fig. 11(a), *L*_*0*_ remains significant up to a certain value of *λ*_*ap*_ (which is larger for larger *U*) after which it rapidly decreases to almost zero. The vanishing of *L*_*0*_ corresponds to the formation of immobile bipolarons corresponding to the CDW phase. From this, we can conclude that large *U* extends the width of SDW phase and narrows down the width of the metallic region. On the other hand, smaller values of *U* effectively widen the metallic region. For a given *U*, *L*_*0*_ is suppressed by the *e-p* interaction strengths **(**dotted curves). (ii) The renormalized hopping integral in Fig. 11(b), does show somewhat similar features as we observe for *L*_*0*_ except that it does not go to zero in the considered range of *λ*_*ap*_. At low anharmonicity, for a given *g*, *t*_*eff*_/*t* drops rather sharply with reduction in *U*, while for a given *U*, *t*_*eff*_/*t* decreases rapidly with *g* at large values of *g*. (iii) Fig. 11(c) shows that the effective *e-e* interaction decreases with increasing *λ*_*ap*_ for all cases of (*α*, *U*) and changes sign at some values of *λ*_*ap*_. For a given *g*, such values of anharmonicity get shifted to lower values as *U* is reduced. For a given *U*, as *g* decreases, the *λ*_*ap*_ value at which *U*_*eff*_ changes sign increases. It is known that interplay between the *e-p* and *e-e* interaction strengths decides the GS of the system. In addition to this, inclusion of the phononic anharmonicity also plays a vital role in determining the GS of the system especially by enhancing the *e-p* interaction strength.

Before we close this section, we would like to point out that Freeeicks and collaborators^53–56^ have studied the Holstein model with anharmonic phonons in some detail. For example, Freericks, Jarrell and Mahan have considered the Holstein model with quartic phonon anharmonicity and solved it in the infinite-dimensional limit using the Quantum Monte Carlo method. They have found that at half-filling the system orders in a commensurate CDW phase and there is no evidence of the system evolving into the superconducting phase. However they have pointed out that with the increase in anharmonicity there could be a possibility of superconductivity prevailing over the CDW order. In the strong-coupling limit, CDW is found to be preferred state because of the band narrowing effect. In our work with *U* = 0, we also find that the system goes from the metallic phase to the CDW phase as the *e-p* interaction is increased. We have also observed that our results are close to the harmonic case at small and large anharmonicities in agreement with the results of Freericks and Zaltic^55, 56^.

## Conclusion

In conclusion, we have considered the Holstein-Hubbard chain with phonon anharmonicity at half filling. Using a series of canonical transformations followed by an averaging with respect to a linear superposition of many-phonon states and the Bethe-ansatz method we obtained the exact GS energy solution of the effective electron Hamiltonian. From the study of local moment formation, double occupancy, Mott criterion and the von Newman entropy, we suggest the existence of an intervening metallic phase at the SDW-CDW cross-over region and also show that low anharmonicity widens this metallic phase. Though it seems it is most likely that a metallic state phase may exist, it may be premature to suggest that this metallic state would be superconductive.
